# Supplementary material for: A Highly Selective and Sensitive Nano-Silver sol Sensor for Hg2+ and Fe3+: Green Preparation and Mechanism
Source: Polymers (Basel). 2022 Sep 7;14(18):3745. doi: 10.3390/polym14183745 (PMC9504428; doi:10.3390/polym14183745)
Supplement: Supplementary file 1 [file polymers-14-03745-s001.zip › polymers-1862811-supplementary.pdf]

polymers
MDPI

Supplementary Material

A Highly Selective and Sensitive Nano-Silver Solutions Sensor for Hg^{2+} and Fe^{3+}: Green Preparation and Mechanism

Yining Yang [1], Xiaodong Zhou [1], Ruitao Dong [1], Yanwei Wang [1], Zichao Li [2], Yun Xue [1,*] and Qun Li [1]

Table S1. Comparison of the proposed Hg^{2+} detection method with other reported methods.

Methods	Probe	Linear range/(μM)	LOD/nm	Ref.
Colorimetric	SSA-Ag NPs	0–5	14	[1]
Colorimetric	AuNPs	0.1–100	0.05	[2]
Electro chemistry	Ag NPs	5.0–755	8.43	[3]
Colorimetric	Ag NPs	0.5–5.0	58.11	[4]
Colorimetric	SA-Ag NPs	0.025–60	5.29	[5]
Fluorescence	NCDs-RhB@COF	0.048–10	15.9	[6]
Colorimetric	CMS/PVP-Ag NPs	0–20<br>20–100	7.1	This work

Table S2. Comparison of the proposed Fe^{3+} detection with other reported methods.

Fluorescent Probe	Linear range/(μM)	LOD/(μM)	Ref.
LCQDs	50–650	0.196	[7]
NP-CQDs	0.05–200	0.05	[8]
S-C-dots	0-872	0.56	[9]
C-QDs	2–50	1.3	[10]
FNCDs	2–25	0.9	[11]
S-doped C-dots	1–500	0.1	[12]
Hyperbranched polyi-mide	0–600	60.6	[13]
MgO	50–500	23	[14]
$PGMN_2$	40–240	1.29	[15]
CMS/PVP-Ag NPs	0–60	0.0036	This work

Reference

1. S. Das, M.N. Aktara, N.K. Sahoo, P.K. Jha, M. Hossain, Sensitive and robust colorimetric assay of Hg2+ and S2− in aqueous solution directed by 5-sulfosalicylic acid-stabilized silver nanoparticles for wide range application in real samples, Journal of Environmental Chemical Engineering, **2017**, *5*, 5645–5654.
2. J.L. Chen, P.C. Yang, T. Wu, Y.W. Lin, Determination of mercury (II) ions based on silver-nanoparticles-assisted growth of gold nanostructures: UV-Vis and surface enhanced Raman scattering approaches, Spectrochimica acta. Part A, Molecular and biomolecular spectroscopy, **2018**, *199*, 301–307.
3. E. Eksin, A. Erdem, T. Fafal, B. Kıvçak, Eco-friendly Sensors Developed by Herbal Based Silver Nanoparticles for Electrochemical Detection of Mercury (II) Ion, Electroanalysis, **2019**, *31*, 1075–1082.
4. A.S. Ertürk, Biosynthesis of Silver Nanoparticles Using Epilobium parviflorum Green Tea Extract: Analytical Applications to Colorimetric Detection of Hg2+ Ions and Reduction of Hazardous Organic Dyes, Journal of Cluster Science, **2019**, *30*, 1363–1373.
5. F. Faghiri, F. Ghorbani, Colorimetric and naked eye detection of trace Hg(2+) ions in the environmental water samples based on plasmonic response of sodium alginate impregnated by silver nanoparticles, Journal of hazardous materials, **2019**, *374*, 329–340.
6. L. Guo, Y. Song, K. Cai, L. Wang, "On-off" ratiometric fluorescent detection of Hg(2+) based on N-doped carbon dots-rhodamine B@TAPT-DHTA-COF, Spectrochimica acta Part A: Molecular and biomolecular spectroscopy, **2019**, *227*, 117703.
7. X. Gao, X. Zhou, Y. Ma, T. Qian, C. Wang, F. Chu, Facile and cost-effective preparation of carbon quantum dots for Fe3+ ion and ascorbic acid detection in living cells based on the "on-off-on" fluorescence principle, Applied Surface Science, **2019**, *469*, 911–916.
8. Y. Guo, F. Cao, Y. Li, Solid phase synthesis of nitrogen and phosphor co-doped carbon quantum dots for sensing Fe3+ and the enhanced photocatalytic degradation of dyes, Sensors and Actuators B: Chemical, **2018**, *255*, 1105–1111.

9. V.M. Naik, D.B. Gunjal, A.H. Gore, S.P. Pawar, S.T. Mahanwar, P.V. Anbhule, G.B. Kolekar, Quick and low cost synthesis of sulphur doped carbon dots by simple acidic carbonization of sucrose for the detection of Fe3+ ions in highly acidic environment, Diamond and Related Materials, **2018**, *88*, 262–268.
10. M. Zhou, Z. Zhou, A. Gong, Y. Zhang, Q. Li, Synthesis of highly photoluminescent carbon dots via citric acid and Tris for iron(III) ions sensors and bioimaging, Talanta, **2015**, *143*, 107–113.
11. R. Atchudan, T. Edison, K.R. Aseer, S. Perumal, N. Karthik, Y.R. Lee, Highly fluorescent nitrogen-doped carbon dots derived from Phyllanthus acidus utilized as a fluorescent probe for label-free selective detection of Fe(3+) ions, live cell imaging and fluorescent ink, Biosens Bioelectron, **2018**, *99*, 303–311.
12. Q. Xu, P. Pu, J. Zhao, C. Dong, C. Gao, Y. Chen, J. Chen, Y. Liu, H. Zhou, Preparation of highly photoluminescent sulfur-doped carbon dots for Fe(iii) detection, Journal of Materials Chemistry A, **2015**, *3* 542–546.
13. A. Xing, X. Miao, T. Liu, H. Yang, Y. Meng, X. Li, An intrinsic white-light-emitting hyperbranched polyimide: Synthesis, structure–property and its application as a "turn-off" sensor for iron(iii) ions, Journal of Materials Chemistry C, **2019**, 7, 14320–14333.
14. A. Jain, S. Wadhawan, V. Kumar, S.K. Mehta, Colorimetric sensing of Fe3+ ions in aqueous solution using magnesium oxide nanoparticles synthesized using green approach, Chemical Physics Letters, **2018**, *706*, 53–61.
15. T. Tang, J. Wang, D. Xu, Synthesis of a novel hyperbranched polymer and its application in multi-channel sensing Fe3+, Research on Chemical Intermediates, **2019**, *46*, 1425–1435.
